# Preserving sequence annotations across reference sequences

**DOI:** 10.1186/2041-1480-5-S1-S6

**Published:** 2014-06-03

**Authors:** Zuotian Tatum, Marco Roos, Andrew P Gibson, Peter EM Taschner, Mark Thompson, Erik A Schultes, Jeroen FJ Laros

**Affiliations:** 1 Department of Human Genetics, Center for Human and Clinical Genetics, Leiden University Medical Center, Einthovenweg 20, 2333 ZC Leiden, the Netherlands; 2 Informatics Institute of the Faculty of Science, University of Amsterdam, Science Park 904, 1098 XH Amsterdam, the Netherlands; 3 Leiden Genome Technology Center, Leiden University Medical Center, Einthovenweg 20, 2333 ZC Leiden, the Netherlands; 4 Department of Rheumatology, Leiden University Medical Center, Albinusdreef 2, 2333 ZA Leiden, the Netherlands

## Abstract

**Background:**

Matching and comparing sequence annotations of different reference sequences is vital to genomics research, yet many annotation formats do not specify the reference sequence types or versions used. This makes the integration of annotations from different sources difficult and error prone.

**Results:**

As part of our effort to create linked data for interoperable sequence annotations, we present an RDF data model for sequence annotation using the ontological framework established by the OBO Foundry ontologies and the Basic Formal Ontology (BFO). We defined reference sequences as the common domain of integration for sequence annotations, and identified three semantic relationships between sequence annotations. In doing so, we created the Reference Sequence Annotation to compensate for gaps in the SO and in its mapping to BFO, particularly for annotations that refer to versions of consensus reference sequences. Moreover, we present three integration models for sequence annotations using different reference assemblies.

**Conclusions:**

We demonstrated a working example of a sequence annotation instance, and how this instance can be linked to other annotations on different reference sequences. Sequence annotations in this format are semantically rich and can be integrated easily with different assemblies. We also identify other challenges of modeling reference sequences with the BFO.

## Background

### Sequence annotations and their relationship with reference sequences

Sequence annotations are information artifacts that add biologically meaningful information to specific locations on genomic, gene, transcript or protein sequences. For example:

1) Gene *OR4F5 *is located on human chromosome 1 (build hg19), from position 69090 to 70008.

2) Substitution of C by T at location 178 of transcript reference sequence *NM_004006.2 *results in nonsense variant Gln60* in protein reference sequence *NP_003997.1*.

Sequence annotations are only meaningful if the reference sequence is known. However, specifying a stable reference is not necessarily straightforward. Before the Human Genome Project, *Locus Specific Databases *(LSDB) were recommended for storing and sharing gene centric variant annotations [1]. To date, the most popular platform for storing these transcript variants is the *Leiden Open-source Variation Database v.2 *(LOVD2) [2]. In each LOVD2 instance, a "stable" transcript sequence is chosen as the reference sequence of each gene. Variants are annotated with descriptions of sequence variations and positions according to the chosen transcript sequence. There are many advantages of using gene/transcript centric annotation approach. First, the length of a gene is much shorter than a locus/chromosome, therefore maintaining the sequence content is much easier. Secondly, it limits annotations mainly to the protein coding regions of the genome, therefore focusing more on easy to predict phenotypic effects. However, LSDBs typically limit descriptions of DNA variants to a single transcript, even when multiple transcripts may be affected. Depending on which transcript is used, the variant description may look very different. To calculate the location of a variant based on a different reference sequence, an external conversion tool has to be used for the position conversion [3]. Disambiguation of the variant description is an essential step in the context of data integration and preservation.

However, not all biological questions are locus specific. As sequencing technologies advanced in the past 15 years, more and more studies are omics focused, requiring a "stable" and "complete" reference genome [4]. The Human Genome project was completed in April 2003, followed by the release of human genome assembly NCBI35/hg17 in May 2004. Sequence gaps and assembly errors were removed and newly discovered genes, (non-coding) transcripts and proteins were annotated with every new release up to GRCh37/hg19 (February, 2009) [5]. As reference sequences are revised, it becomes increasingly difficult to track and compare annotations. Researchers today share their results of genome-wide genomic and epigenetic studies in publications and databases, but they often fail to mention the exact version of the reference genome sequence. Moreover, many popular annotation file formats do not explicitly ask for reference sequence version information. It is up to the user to embed this information in the file description through natural language. Consequently, when using these formats to exchange data for computational analysis and data integration, essential metadata is too easily lost. For example, the ENCODE Project Consortium [6] has effectively shared their data by publishing them as annotation tracks in the UCSC genome browser [7]. However, these annotation tracks use Browser Extensible Data (BED) format, which does not explicitly state the reference assembly version within the file. To propagate current annotations to the forthcoming GRCh38/hg20 and alternative genome assemblies, it is crucial to preserve annotations with their respective reference sequence versions.

### A Semantic Web approach to data integration

A possible approach to exposing sequence variation annotations in a computer accessible format is provided by Sematic Web languages and tools [8]. It effectively removes the boundaries between annotating data, linking data, and making data machine readable [9-11]. By representing data and metadata in *Resource Description Framework *(RDF) and using shared ontologies in RDF and *Web Ontology Language *(OWL), mismatches between database schema's and the identity of its content can be addressed [12,13]. A first attempt for mutation data was presented by Zappa and coworkers, who produced a mutation database for *TP53 *as Linked Open Data [14]. They followed the principles of Linked Data [15] and applied various existing ontologies to achieve optimal interoperability. However, they did not address the problem of integrating mutation data that were annotated using different reference sequences. They did not model genomic locations of annotations in detail, which makes querying this dataset difficult.

### Ontological framework for data integration across resources

Formal ontologies play an important role in semantic data integration between information systems [16,17], bringing conceptual coherence, stability, and scalability to the applied domain, which can greatly increase data interoperability [17,18]. The *Open Biological and Biomedical Ontologies *(OBO) Foundry provides a suite of orthogonal interoperable ontologies to aid knowledge integration in the biomedical domain [19]. To take advantage of the OBO Foundry ontologies, we have chosen *Basic Formal Ontology *(BFO) [20] as our upper ontological framework for data modeling [20]. Other ontologies in OBO that are relevant to this paper include the *Information Artifact Ontology *(IAO) [21], the *Sequence Ontology *(SO) [22], the *Ontology for Genetic Interval *(OGI) [23], and the *Relation Ontology *(RO) [24].

Previous efforts on modeling biological sequences and sequence annotations in the OBO community have taken primarily a biological viewpoint. Thus, sequences refer to biological molecules, and sequence annotations refer to features defined with respect to biological process [22,25]. The SO focuses on creating a set of consistent vocabularies that describe the biological functions of these sequences and defining the biological relationships between these sequences [22]. OGI models the biological physical sequence by adopting the realism approach from BFO, and further contributes to this model by adding spatial topological relationships between sequences [23]. However, Hoehndorf et al. pointed out a gap between this biological model and information systems that are used to store sequence annotations [26]. To bridge this gap, they have proposed three views of biological sequences: molecular, syntactic, and abstract. *Molecular sequences *are DNA and RNA molecules as well as proteins. *Syntactic sequences *are strings like "ACAC" and represent the arrangement of the molecules in the molecular sequences. *Abstract sequences *represent an equivalence class of sequence tokens or representations. They point out that without such a clear distinction data integration is hampered. Indeed, the SO community acknowledged the lack of distinction that is made by biologists between abstract, syntactic, and molecular sequences. Bada and Eilbeck proposed a strategy of separating SO into two parallel ontologies: one for molecular sequences, the other with abstract sequences (abstract in a broader sense than meant by Hoehndorf). The former would be an extension of the Molecular Sequence Ontology while the SO would focus more on the abstract sequences referring to sequences, and parts of sequences [27]. However, this new alignment strategy is still under discussion.

Beyond the OBO Foundry there are additional relevant ontologies applicable to sequence annotation. The *Feature Annotation Location Description Ontology *(FALDO) is the latest effort to address the void of describing sequence annotations from the information systems' perspective [28]. It is designed to be general enough to describe annotations with various level of location complexity, but not addresses issues such as the meaning of or the evidence of the location.

### Aim of this paper

Our aim is to create an RDF data model for describing sequence annotation instances within an established ontological framework that fits our practice of working with reference sequences and different versions of genome assemblies. We provide a mechanism for linking annotation instances to different reference sequences. We also present some of the challenges in aligning our approach with current OBO Foundry ontologies.

## Results and discussion

### Describing sequence annotation instances

**Figure 1:**
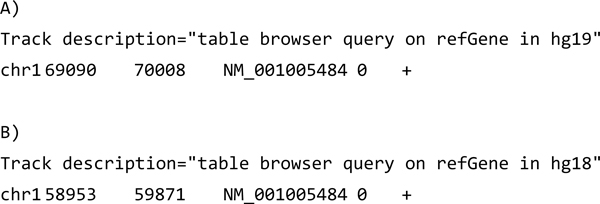
**BED file examples**. RefSeq transcript annotation in BED format on genome builds hg19 (a) and hg18 (b). The second line contains the start and end positions of the *NM_001005484 *transcript encoded by the *OR4F5 *gene that differ per assembly. Note that the BED file header line does not explicitly state the reference sequence information. The submitter can only embed this information in the track description through natural language.

Our starting point for modeling sequence annotations was the BED format, a widely used table-based format for sequence annotations that is easy to use and efficient to store (see Figure 1). It typically consists of rows with a reference (e.g. a chromosome identifier), start and end position on that reference, and a value for the annotation. Most UCSC genome browser annotations can be downloaded as BED tracks. We started by deriving our RDF model from the BED format: (i) we identified the desired upper ontological framework for the domain of interest; (ii) we converted data in the BED track to RDF triples; (iii) we further transformed the resulting triples by adding class definitions and ontology mappings to the final model. We describe these steps below:

#### Upper ontological framework

We chose to use the BFO (version 1.1) as our top-level ontological framework. We augmented BFO with a minimal *Reference Sequence Annotation *(RSA) ontology to capture classes and predicates, and defined alignment strategies for RSA with OBO.

#### Data transformation to triples

**Figure 2:**
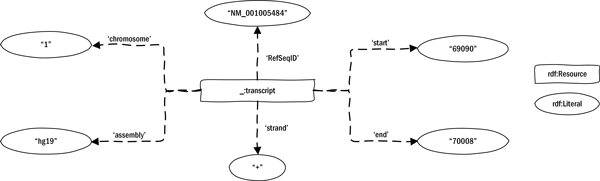
**Naive model**. Naïve transformation of a BED sequence annotation. Predicates used in this model are placeholders and replaced in a later stage.

**Figure 3:**
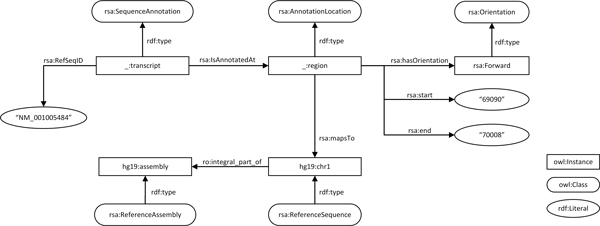
**Semantic model**. A sequence annotation instance after semantic transformation.

As a preparative step, we first created annotation instances that closely matched our original data format. We created a 'naive' model for sequence annotation to directly translate the information in the BED file with the addition of the reference assembly name (Figure 2). Predicates linking the resource and its property values were derived from the BED format description. At this stage, we used *rdfs:Literal *to capture concepts without further ontological grounding (i.e., *rdf:type *relations). This data-centric approach to semantic modeling is similar to the 'syntactic' conversion that is often used for integration of non-RDF resources, where table values are converted to literals, and table names and headers to classes and properties without any further semantic modelling [29]. These naive models usually have limited semantic depth, such that finding common elements for integration with other data sources can be difficult. Therefore, the model is often linked to a more sophisticated, or personal model. In our case, we used the naive model as a starting point in the modeling process, replacing it step by step by a more precise model (Figure 3). Content of *rdfs:Literals *from the naive model were thus converted to *owl:instances*, and class definitions were added. Below, we discuss our derivation of the new model step-by-step, while explaining the placement of new RSA classes and predicates, the reuse of existing ontologies, and potential problems with OBO alignment. An RDF representation of the final model is shown as follows:

@prefix xsd: <http://www.w3.org/2001/XMLSchema#> .

@prefix rsa: <http://rdf.biosemantics.org/ontologies/rsa#> .

@prefix hg19: <http://rdf.biosemantics.org/data/genomeassemblies/hg19#> .

@base <http://rdf.biosemantics.org/examples/sequence_annotation#> .

:transcript a rsa:SequenceAnnotation ;

    rsa:refseqID "NM_001005484";

    rsa:isAnnotatedAt :location .

:location a rsa:AnnotationLocation ;

    rsa:start "69090"^^xsd:int ;

    rsa:end "70008"^^xsd:int ;

    rsa:mapsTo hg19:chr1 ;

    rsa:hasOrientation rsa:forward

hg19:chr1 a rsa:ReferenceSequence ;

    ro:integral_part_of hg19:assembly .

hg19:assembly a rsa:ReferenceAssembly .

#### Modeling locations on a reference sequence

We considered two approaches to harmonizing genomic location across different reference sequences. On the one hand, one may consider the location as an integral part of the annotation. That is, if the location is changed, the annotation becomes a different annotation. For example, variant annotations generally include the location of the annotation as part of the identifier. Thus, the change of location results in change of identifier of the annotation. On the other hand, a location can be considered an instance separate from the annotation. In this way, a single annotation can be associated with multiple locations and a single location can be associated with multiple annotations. In our example, the second approach is more appropriate because it provides a mechanism to link an annotation to locations on different reference sequences and sequence assemblies. Therefore, we created an instance of *RSA:AnnotationLocation :region*, as the subject of positional properties. We defined the instance of *hg19:assembly *and *hg19:chr1 *as *ro:integral_part_of hg19:assembly*. We linked *:region *to *hg19:chr1*, which indirectly linked this annotation with the reference assembly.

In the example shown in Figure 3, we kept the *:start *and *:end *as *rdfs:Literals*. It is also possible to convert the values of *:start *and *:end *to *rdfs:Resource*, and assign values to these resources. However, we argue that *:start *and *:end *should be treated as data type properties of a region. By doing so, we discourage linking of other RDF resources to *:region *boundaries and the smallest linkable resource remained to be *:region*. Furthermore, in practice, using *rdfs:Resource *to describe the start and end of a region (simply two numbers) leads to an explosion of triples. Hence, our model expresses instance data in its simplest form. In contrast, FALDO defines *:start *and *:end *as instances of *faldo:Positions*. It uses more triples (12 instead of 2) to describe the two points. A benefit of FALDO's approach is that it gives more flexibility to describe fussy regions.

#### Model strand-ness of sequence features

In contrast to RNA and protein, the stranded-ness of DNA sequences needs to be addressed when modeling DNA sequence annotations. Because the two DNA strands are the reverse complement of each other, information encoded in one orientation can be derived from the other strand. Consequently, sequence records in DNA databases contain only one of the two DNA strands (as the other stand can be inferred), but this does not necessarily mean this is the strand an annotation pertains to. We have to take this into account when modeling the strand in an annotation.

When annotations are only linked to the reverse strand of a reference sequence, there are two conceptual annotation models. "Reverse strand annotations" can be understood either as annotations on a sequence that is the reverse complement of a reference sequence, or they can be understood as annotations on the reference sequence, but their interpretations are based on the reverse complement. In the first conceptualization, we need to link an annotation instance to a new sequence instance that is the reverse complement of a known reference sequence. In the second conceptualization, the reverse-ness is a quality of the annotation similar to length being a quality of a region. In practice, most sequence annotation systems specify coordinates using one strand as reference (the forward strand) and "strand" or "orientation" to indicate which strand an annotation pertains to. Thus, the stranded-ness in our example data refers to how annotations can be interpreted on single strand sequences. We modeled this in our example RDF with *:hasOrientation :forward*.

We further argue that orientations of annotated regions are not limited to *:forward *and *:reverse*. If an annotation represents a sequence feature of both strands, such as a CpG Island, we consider the orientation of an annotation as *:bidirectional*. If the reference sequence is a *syntactic sequence *representing single strand molecules (RNA, protein), or if the sequence feature does not rely specifically on the underlying sequence (as in the case of a specific binding or chromatin features), the annotation orientation is *:none*. As a result, the class for annotation orientation is defined as an enumeration of four disjoint instances.

RSA:Orientation subclass of

    { RSA:forward, RSA:reverse, RSA:bidirectional, RSA:none } .

#### RSA classes and alignment with OBO

We have created instances using five classes from RSA. To enable better integration of our data to existing linked data, we considered how to align RSA classes with OBO classes.

*RSA:SequenceAnnotation *can be regarded as an *SO:sequence_feature *with an annotated location on a reference sequence. However, SO is currently not directly aligned with BFO, although this is an ongoing effort [25]. To further improve the consistency and interoperability of SO, new approaches to the BFO alignment were proposed. Terms in SO could be distinguished as either molecular sequences (*BFO:independent_continuant*, IC) or abstract sequences (*BFO:generically_dependent_continuant*, GDC) representing molecular sequences [27]. This distinction provides a foundation for the alignment between SO and BFO. Following the same alignment strategy, we chose to refer to *SO:sequence_feature *as a subclass of GDC. While it is not necessarily true that all terms under *SO:sequence_feature *can be GDCs, it is outside the scope of this paper to define which section of *SO:sequence_feature *falls into GDC. Because *RSA:SequenceAnnotation *is an information entity, we also considered to use *IAO:information_content_entity *as its super class. However, it is not clear to us whether a class can be the subclass of both *SO:sequence_feature *and *IAO:information_content_entity*, because the definition of *SO:sequence_feature *under GDC is still under discussion. We therefore defer the alignment between *RSA:SequenceAnnotation *and *IAO:information_content_entity *to the alignment between SO and IAO. Meanwhile, IAO provides a useful link between database row instances and annotation instances. For example, an instance of *RSA:SequenceAnnotation *can be the object of *IAO:is_about*.

To summarize, we defined *RSA:SequenceAnnotation *as a subclass of *BFO:generially_dependent_continuant*, and in particular,

RSA:SequenceAnnotation subclass of

    SO:sequence_feature and

    RSA:isAnnotatedAt some RSA:AnnotationLocation

*RSA:AnnotationLocation *is a constraint on a reference sequence in terms of location and orientation, with data properties such as a start point and an end point. We argue that it should be classified as a GDC in BFO, because it cannot exist outside the context of an annotation of a reference sequence. However, this prevents alignment with other relevant classes in OBO. For instance, *OGI:Biological_interval *provides the location properties and it defines relationships between two instances of intervals such as by *OGI:isLocatedBefore*. Nevertheless, *OGI:Biological_Interval *is defined as the "spatial continuous physical entity" and a subclass of *BFO:object*, and thus a subclass of IC. In the context of sequences, this defines an interval as a molecular sequence. Therefore, we only defined relationships between the orientation, the reference, and the annotation location in the scope of RSA.

RSA:AnnotationLocation subclass of

    RSA:hasOrientation some RSA:Orientation and

    RSA:mapsTo some RSA:ReferenceSequence

*RSA:ReferenceSequence *is about biological sequences, and modeling biological sequences in ontologies is not easy [26]. In RSA, we defined *RSA:ReferenceSequence *as a *syntactic sequence*. This is an information-bearing entity that contains a series of letters from a given alphabet (i.e., ATGC for DNA). It can represent sequential information captured by a biological molecule, but may represent a (possibly empty) set of molecules. It can be stored in computer systems or on a piece of paper, therefore its physical existence is an instance of *IAO:information_content_entity*. To correctly model reference sequences, it is crucial to distinguish between the sequence content and the file storing the sequence content, and therefore define *RSA:ReferenceSequence *not a subclass of *IAO:information_content_entity*. For example, both transcript sequences and chromosome sequences can be used as reference sequences, so instances of *RSA:ReferenceSequence *can be *ro:proper_part_of *another instance. This *part of *relationship is important for data integration scenarios shown in the next section, and this *part of *relationship works only if *RSA:SequenceAnnotation *is defined by the sequence content, as the sequence content of a transcript can be *part of *the sequence content of a chromosome. However, if *RSA:ReferenceSequence *is defined as a subclass of *IAO:information_content_entity*, the *part of *relationship cannot be modeled because the file of a transcript sequence is not part of the file of the chromosome sequence.

In addition, we were confronted with the limitations of the reality constraint of BFO [30]. In the field of sequence annotations, biologists often work with abstract entities that only have an indirect relation to entities that exist in reality. For instance, the notion of a consensus sequence is widely used in practice. Consensus sequences are hypothetical sequences designed to capture information not from single molecules, but from sets similar molecules. In the case of reference sequence modeling we must accommodate consensus sequences. If we modeled *RSA:ReferenceSequence *as a subclass of GDC, the instance *hg19:chr1 *(chromosome 1 in human genome assembly version 19) *inheres in *an instance of a corresponding molecular sequence. However, there is no molecular sequence that corresponds with the sequence content of *hg19:chr1*, because *hg19:chr1 *is the consensus of the sequence content of chromosome 1 of multiple people. The consensus sequence modeling problem not only applies to sequences in genome assemblies, but also to all sequences generated by Next Generation Sequencing technologies. Even in the context of personal genome sequencing, a sequence may not be derived from a single molecule from a single cell, but from a set of molecules from multiple cells. As discussed by Hoehndorf *et al*., proper definitions of biological sequences require the upper ontological framework to handle hypothetical sequences [26]. Thus, we argue that how to define a consensus sequence within the framework of BFO and OBO needs to be addressed by the OBO community. SO provides class *SO:consensus_region *for consensus sequences. However, this class is not aligned with BFO, and it is unclear whether this class is designed with OBO principles.

Finally, *RSA:ReferenceAssembly *is an information entity encapsulating a set of *RSA:ReferenceSequence*s that are often used together to represent the total sequence content of an organism, and *RSA:ReferenceSequence *is *RO:proper_part_of RSA:ReferenceAssembly*. Its version number (in some cases, the timestamp) is crucial for data integration. *RSA:ReferenceAssembly *cannot be aligned with BFO, because its parts are not aligned with BFO.

### Semantic relations between annotations

**Figure 4:**
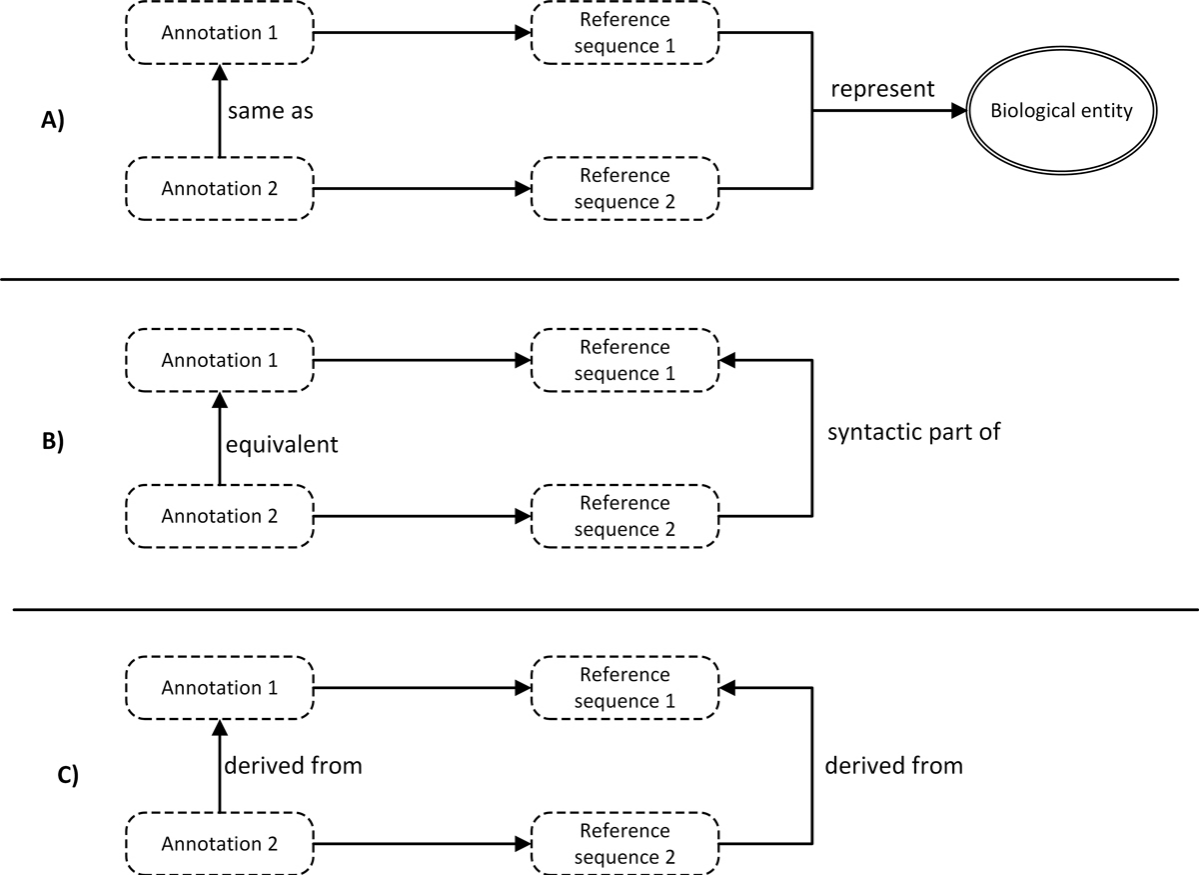
**Semantic relationships between annotations**. The sematic relationship between annotations is determined by the relationship between their reference sequences. 1) An annotation is the same as another annotation if their reference sequences represent the same biological entity. 2) An annotation is equivalent of another if its reference sequence is syntactic part of the other reference sequence. 3) An annotation is derived from another if it reference sequence is derived from the other reference sequence.

With a complete ontological framework in place, we then investigated how sequence annotations using different reference sequences can be semantically linked. Semantic relationships between sequence annotations are determined by the relationship between their reference sequences. We categorized three types of reference sequence relationships that are crucial for data integration: 1) The two reference sequences represent the same biological entity; 2) One reference sequence is a syntactic part of the other reference sequence; 3) One reference sequence can be syntactically derived from the other reference sequence. Here, we show how each reference sequence relationship defines the relationship between annotations in Figure 4.

The *same as *relationship is important for integrating annotations based on different reference assemblies (Figure 4A). For example, the gene annotation of *OR4F5 *based on hg19 is the same as the one based on hg18. We note that the properties of the underlying reference sequence may differ, and hence the two annotations may have different properties (the start and end points on chromosome 1), but they share the same identifier (*OR4F5*). The *equivalent *relationship is important for integrating annotations with different sequence features (Figure 4B). For example, the variant annotation *NM_004006.2:c.178C>T *is equivalent to variant annotation *NC_000023.10:g.32867853G>A*. Although these two variant annotations are defined by different positions and different nucleotide substitutions, they describe the same biological variation from two different viewpoints. The *c*. notation uses transcript as the reference sequence and captures the effect of variation on RNA, whereas the *g*. notation uses chromosome as the reference sequence, and captures the effect on the genome. The *derived from *relationship is important for connecting annotations that occurred in different biological processes (Figure 4C). For example, variant annotation on the protein level NP_003997.1:p.Gln60* is derived from variant annotation on the transcript level *NM_004006.2:c.178C>T*.

### Interoperability across reference assemblies

To define the relationship between reference sequences from different reference assemblies is not trivial. In line with semantic data integration strategies [29], our goal was to define the common domain of integration across reference assemblies at the chromosome level. However, this domain of integration is outside the scope of RSA. Modeling the relation between consensus sequence and chromosome in line with BFO was not straightforward. In this section, we present three possible methods to connect reference sequences across assemblies.

**Figure 5:**
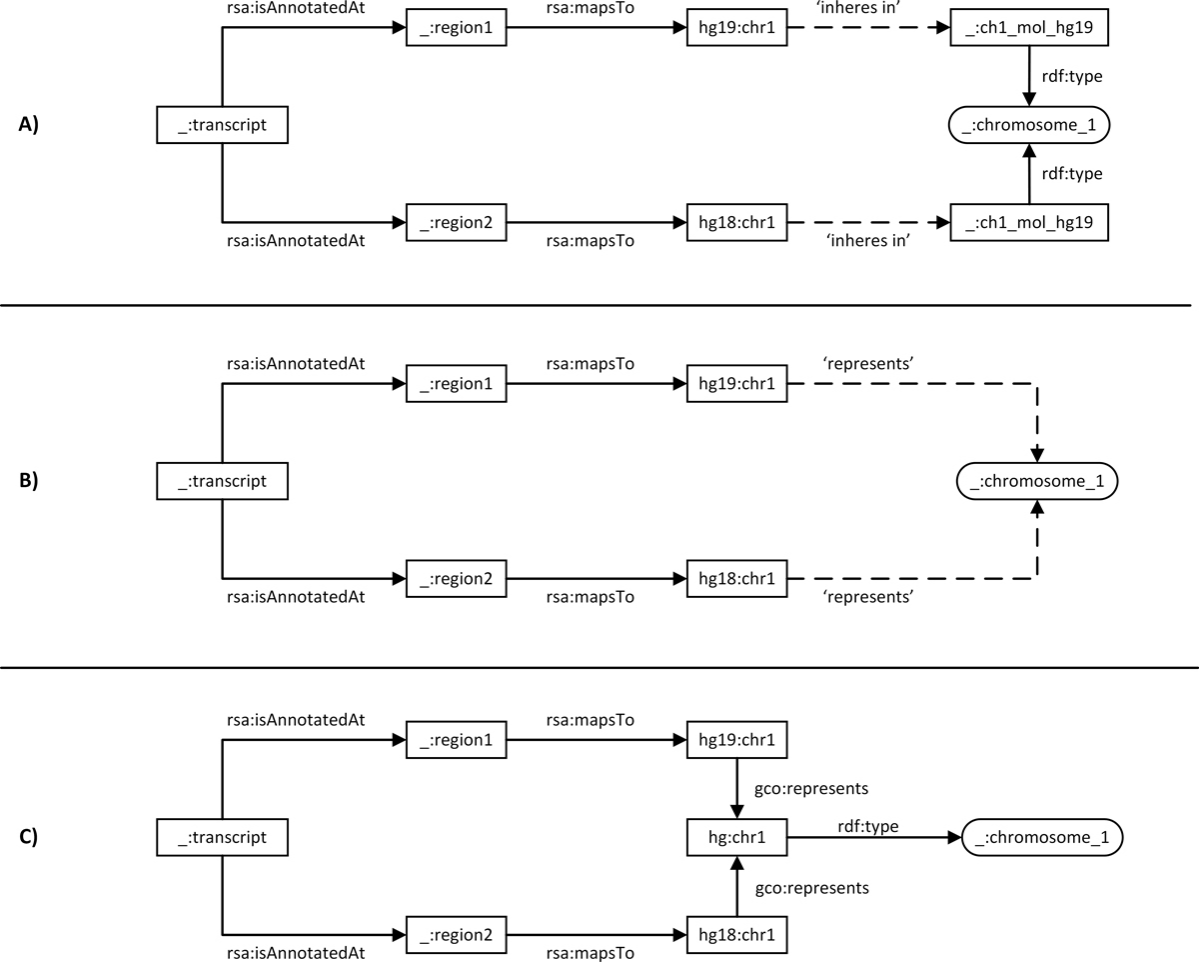
**Domain of integration models**. Three ways to link reference sequences. A) Linking two reference sequences via two molecule instances of the same class. B) Linking two reference sequences via a single class. C) Linking reference sequences via an instance of abstract chromosome.

The first method uses the 'inheres in' property to relate the two instances of class Chromosome 1 that then represent the common domain (Figure 5A). This approach seems to follow BFO. However, we did not find an existing superclass for Chromosome 1, because *hg19:chr1 *and *hg18:chr1 *are consensus sequences that do not inhere in any particular chromosome. The superclass for Chromosome 1 would require an equivalent of a consensus chromosome that is a subclass of IC, which we have shown in the last section is not currently possible.

The second method is perhaps the least attractive, because it defines a relationship between an *OWL:Individual *and an *OWL:Class *that is not a class assertion, violating the OWL-DL definition and making reasoning over datasets undecidable. However, this method eliminates the need for 'real chromosome instances' required by the 'inheres in' relationship in the first method (Figure 5B).

The third method uses a single instance of an abstract class Chromosome 1 (Figure 5C), using the *Genome Component Ontology *(GCO). GCO does not follow BFO's realism viewpoint, and is intentionally kept as minimal as possible. It defines the abstract division of the total genetic information of an organism by its physical separation into different components, but not to describe any specific characteristics derived through experimentation. Instances of *GCO:GenomeComponent *provide high level references. More specific descriptions, such as gene content, length, function, location, loci or sequence, can be linked to instances representing instances of *GCO:GenomeComponent*.

Each method has advantages and disadvantages. We consider method 3 the best option for data integration, because it offers good features for linking and integrating data without violating OWL-DL restrictions. A disadvantage is that it is not aligned with BFO, which may impede integration with data annotated using a BFO-based ontology. Therefore, we retained only the minimal set of classes in GCO. The RDF representation for the model shown in Figure 4C is accessible at http://rdf.biosemantics.org/examples/gco_integration.

## Conclusions

We demonstrated a working data model of sequence annotations that can be preserved across different reference sequence assemblies. This data model uses the ontology of Reference Sequence Annotation, which is available at http://purl.bioontology.org/ontology/RSA.

We also demonstrated that within the scope of our model, BFO could not accommodate all instances required for our purpose when we followed the realism constraint defined by BFO and restrict instances to OWL-DL. However, we cannot exclude that different viewpoints towards modeling genome annotations can provide new insights that would fit the current BFO. GCO was created as our best effort to provide interoperability across reference assemblies, which is available at http://purl.bioontology.org/ontology/GCO. The alignment between RSA, GCO, and OBO will be an ongoing effort.

## List of abbreviations used

LSDB: Locus Specific Databases; LOVD2: Leiden Open-source Variation Database v.2; ENCODE: ENCyclopedia Of DNA Elements; UCSC: University of California, Santa Cruz; BED: Browser Extensible Data; RDF: Resource Description Framework; OWL: Web Ontology Language; Open Biological and Biomedical Ontologies; BFO: Basic Formal Ontology; IAO: Information Artifact Ontology; SO: Sequence Ontology; OGI: Ontology of Genomic Intervals; RO: Relation Ontology; FALDO: Feature Annotation Location Description Ontology; RSA: Reference Sequence Annotation; IC: BFO:independent_continuant; GDC: BFO:generically_dependent_continuant; GCO: Genome Component Ontology

## Competing interests

The authors declare that they have no competing interests.

## Authors' contributions

ZT conceived of the study; ZT, AG and MT discussed and designed the sequence annotation instances and ontologies; ZT, MR and JFJL discussed and designed integration models; ZT drafted the manuscript; EAS and PT contributed to the manuscript; MR and JFJL supervised the project. All authors read and approved the final manuscript.

## Declarations

The publication costs for this article were funded by the RD-Connect project.

This article has been published as part of *Journal of Biomedical Semantics *Volume 5 Supplement 1, 2014: Proceedings of the Bio-Ontologies Special Interest Group 2013. The full contents of the supplement are available online at http://www.jbiomedsem.com/supplements/5/S1.
